# Anatomical Single-Bundle Anterior Cruciate Ligament Reconstruction Using a Calcium Phosphate-Hybridized Tendon Graft with More than an Average of 5 Years of Follow-Up: A Follow-Up Study of a Randomized Controlled Trial

**DOI:** 10.3390/jcm12134437

**Published:** 2023-06-30

**Authors:** Hirotaka Mutsuzaki, Tomonori Kinugasa

**Affiliations:** 1 Center for Medical Science, Ibaraki Prefectural University of Health Sciences, 4669-2 Ami, Ibaraki 300-0394, Japan; 2 Department of Orthopaedic Surgery, Ibaraki Prefectural University of Health Sciences Hospital, 4773 Ami, Ibaraki 300-0331, Japan; 3 Department of Orthopaedic Surgery, Ichihara Hospital, 3681 Oozone, Tsukuba 300-3295, Japan; yan-k@da2.so-net.ne.jp

**Keywords:** anatomical single-bundle anterior cruciate ligament reconstruction, calcium phosphate hybridization, bone tunnel enlargement, osteoarthritis

## Abstract

Calcium phosphate (CaP)-hybridized tendon grafting using an alternate soaking process improves tendon-to-bone healing in anterior cruciate ligament (ACL) reconstructions. This study aimed to compare bone tunnel enlargement, knee osteoarthritis, and clinical results between CaP-hybridized tendon grafting and conventional grafting in anatomical single-bundle ACL reconstruction. This study was a follow-up of a randomized controlled trial. Between July 2011 and December 2015, 90 patients underwent unilateral anatomical single-bundle ACL reconstructions and were randomly assigned to the CaP-hybridized tendon grafting (CaP group, n = 45; age, 27.1 [14–54] years; sex, 21 males and 24 females) or conventional grafting (control group, n = 45; age, 22.9 [13–58] years; sex, 26 males and 19 females). The randomization was performed according to the days of the week when the patients first visited the outpatient. The CaP-hybridized tendon grafting was created intraoperatively. The tendon grafts were soaked in a calcium solution for 30 s. After that, the tendon grafts were soaked in a NaHPO4 solution for 30 s. This soaking cycle between the calcium solution and the NaHPO4 solution was repeated 10 times. The bone tunnel enlargement, osteoarthritis grade, clinical score, and sports level were evaluated in patients who could be followed up for >3 years (CaP group, *n* = 20, average follow-up period 6.0 [5.1–6.9] years; control group, *n* = 15, average follow-up period 5.6 [4.3–6.9] years). Clinical scores, sports levels, and osteoarthritis grades were analyzed using a generalized linear mixed model (GLMM) based on repeated measurement data from preoperative and final observations, with time, group, sex, age, and BMI as fixed effects and the effect of individual differences as variable effects. In addition, bone-tunnel enlargements were analyzed using generalized linear models (GLM) with group, sex, age, and BMI as the main effects. Compared with the control group, the CaP group exhibited significantly reduced bone-tunnel enlargement on the femoral side (anteroposterior diameter; CaP group, 7.9% [−1.1–16.8] vs. control group, 29.2% [17.9–40.5], *p* = 0.004, MCID 16.05, proximal-distal diameter; CaP group, 7.9% [−1.9–17.8] vs. control group, 22.8% [10.9–34.7], *p* = 0.062, MCID 15.00). The osteoarthritis grades progressed in both groups (*p* < 0.001). The clinical scores and sports levels were not significantly different between the groups. This study suggests that the calcium phosphate-hybridized tendon graft reduces femoral bone-tunnel enlargement after anatomical single-bundle anterior cruciate ligament reconstruction in an average >5-year follow-up period. A longer follow-up period is necessary to reveal the clinical effects of the calcium phosphate-hybridized tendon grafts in anterior cruciate ligament reconstruction.

## 1. Introduction

The anterior cruciate ligament (ACL) is located within the knee joint that connects the femur and tibia [1]. The insertion of the ACL is structured through a cartilage layer when transitioning from the ligament to the bone [1]. The primary function of the ACL is to control the anterior and rotational movement of the tibia in relation to the femur [1]. However, the ACL is susceptible to injury from various traumas, including sports injuries and accidents. When the ACL is injured, it leads to instability of the knee joint [2,3]. ACL reconstruction is commonly used for the treatment of ACL injury [2,3]. Typically, hamstring tendon grafts, such as the semitendinosus tendon or both the semitendinosus and gracilis tendons, are used in ACL reconstruction [2,3]. However, bone tunnel enlargement frequently occurs after ACL reconstruction with hamstring tendon grafts [4,5]. The mechanisms underlying bone tunnel enlargement after ACL reconstruction are associated with a complex interplay between biological and mechanical factors [4]. For example, a mechanically inferior interface with fibrous bonding was reported between the grafted tendon and bone after ACL reconstruction using the hamstring tendon [6,7,8]. In addition, it has been reported that patients undergoing ACL reconstruction using soft tissue graft frequently have postoperative knee osteoarthritis at long-term follow-up [2,9]. These phenomena are believed to be caused by microinstability resulting from micromotion in the grafted tendon-bone tunnel.

To address these issues, we explored the potential of improving tendon-to-bone healing in ACL reconstruction as a means to prevent graft tunnel micromotion and reduce bone tunnel enlargement. To achieve this and enhance tendon-to-bone healing, a technique called hybridization of calcium phosphate (CaP) with tendon grafts using an alternating soaking process was developed [10]. The tendon graft is soaked in a calcium solution for 30 s followed by a phosphate solution for 30 s, and this cycle is repeated 10 times [11,12]. X-ray diffraction analysis revealed the presence of low-crystallinity apatite and dicalcium phosphate dihydrate (DCPD: CaHPO_4_·2H_2_O) in the CaP-hybridized tendon [11,12]. The DCPD disappeared after the CaP-hybridized tendon was immersed in an isotonic 5% glucose solution for 24 h [11,12]. In the CaP-hybridized tendon, needle-like low-crystalline apatite (30–50 nm long) is gradually deposited from the surface to a depth of 200-µm in the tendon graft, as observed using von Kossa staining and transmission electron microscopy [11,12]. The CaP-hybridized tendon contains apatite and type I collagen, resembling the bone’s microstructure. The CaP-hybridized tendons were bonded to the bone for 2–3 weeks after ACL reconstruction in rabbits [11,12]. Osteoblasts and osteoclasts may recognize the CaP-hybridized tendon surface implanted in the bone tunnel as bone and form new bone on the CaP-hybridized tendon surface [11,12]. Mechanical experiments on the CaP-hybridized tendon grafts showed superior results to those of the conventional method at 6 months and 1 year after ACL reconstruction in goats [8,13]. In the CaP group, the anterior translation of the tibia relative to the femur when an anterior force was applied to the tibia was smaller than that in the control group [8], while the in situ force in the tendon graft was greater than that in the control group after ACL reconstruction in goats [8,13]. The interface of the grafted tendon and bone was observed through the fibrous tissue in the control group, whereas in the CaP group, direct bonding between the grafted tendon and bone or bonding through the cartilage layer between the grafted tendon and bone was frequently observed [8,13]. Moreover, tendon-to-bone healing at the joint aperture site was observed more frequently in the CaP than in the control group [8,13]. The improvement of tendon-to-bone healing at the joint aperture site in the CaP group is considered to be related to the superiority of the mechanical test [8,13]. Therefore, the advantages of using the CaP-hybridized tendon in ACL reconstruction lie in the improvement of tendon-to-bone healing and mechanical properties. In contrast, the disadvantage is that the operation time may increase by approximately 10 min due to the creation of the CaP-hybridized tendon during the surgical procedure.

In a clinical trial comparing the CaP group and the control group, it was found that bone tunnel enlargement in the femoral side was smaller in the CaP group 1 year after anatomical single-bundle ACL reconstruction, as compared with the conventional method [14,15]. This suggests that the CaP-hybridized tendon graft promoted bone formation within the bone tunnel and improved tendon-to-bone healing at the joint aperture site, effectively controlling graft tunnel micromotion and reducing bone tunnel enlargement. Moreover, the femoral bone tunnel in the CaP group did not expand or progress over time compared with that in the control group for up to 2 years postoperatively [16]. However, the clinical scores and sports levels did not differ between the groups [14,15,16]. As the participants were followed for 2 years [14,15,16], further investigation with longer follow-up periods is warranted.

Therefore, this study aimed to compare bone tunnel enlargement, knee osteoarthritis, and clinical results between the CaP-hybridized tendon grafts and the conventional method in anatomical single-bundle ACL reconstruction for patients with a >3-year follow-up period. We hypothesized that the CaP-hybridized tendon graft would reduce femoral bone tunnel enlargement, and the clinical results would be similar. Prevention of bone tunnel enlargement owing to the CaP-hybridized tendon grafts may have clinical significance.

## 2. Materials and Methods

This study was a follow-up of a randomized controlled trial. Between July 2011 and December 2015, 90 patients underwent unilateral anatomical single-bundle ACL reconstructions and were randomly assigned to the CaP-hybridized tendon grafting (CaP group, *n* = 45) or conventional grafting (control group, *n* = 45) [14,15,16]. Patients who had revision cases or multi-ligamentous surgeries were excluded from the study [14,15,16]. Randomization was performed based on the patient’s first outpatient visit day. The patients visited the outpatient clinic of the Ichihara Hospital and were diagnosed with ACL injury based on their physical examinations (positive anterior drawer test, positive Lachman test, and positive pivot-shift test findings) and magnetic resonance imaging findings (disruption, elongation, and increased intensity of the ACL). The evaluation was made by orthopedic surgeons with over 10 years of experience. The ACL reconstructions were performed in an inpatient setting, and the patients were discharged after 1–2 weeks, followed by an outpatient examination. In this study, patients who could be followed up for >3 years were evaluated. The evaluations were performed during an outpatient visit. The investigations were conducted based on medical records in January 2023. The ethics committee of the Ichihara Hospital reviewed and approved the study (approval number: 1101). Informed consent was obtained from all enrolled patients. Consent was also obtained from the parents of young patients aged <20 years.

The surgical procedure, intraoperative CaP hybridization method, and postoperative rehabilitation were performed between 2011 and 2015, and the method was provided in the previous publications of this randomized clinical trial [14,15,16].

### 2.1. Clinical Evaluations

Patients’ sports levels were evaluated using the Tegner scale [17] (with scores ranging from 0 [low] to 10 [high] points) before ACL injury and at the final observation. The Lysholm scale, which assesses various aspects, including a limp, support, stair climbing, squatting, instability, pain, swelling, and atrophy of the thigh [17], was used before ACL reconstruction and at the final observation. Furthermore, re-rupture and any adverse events (including cancer, fracture, infection, severe pain, and contracture) were recorded in both groups.

### 2.2. Radiograph Evaluations

Two plain radiographs (front and lateral views) were obtained preoperatively and at the final observation. Knee osteoarthritis was evaluated using the Kellgren–Lawrence (K–L) grading system as follows: Grade 0 (normal knee; no osteophytes or joint space narrowing), Grade 1 (possible osteophytic lipping and doubtful narrowing of joint space), Grade 2 (definite osteophytes and possible narrowing of joint space), Grade 3 (moderate multiple osteophytes, definite narrowing of joint space and some sclerosis, and possible deformity of the bone end), and Grade 4 (large osteophytes, marked narrowing of joint space, severe sclerosis, and definite deformity of bone ends) [18]. The K–L grading was compared between the two groups at the preoperational and final observation.

**Figure 1:**
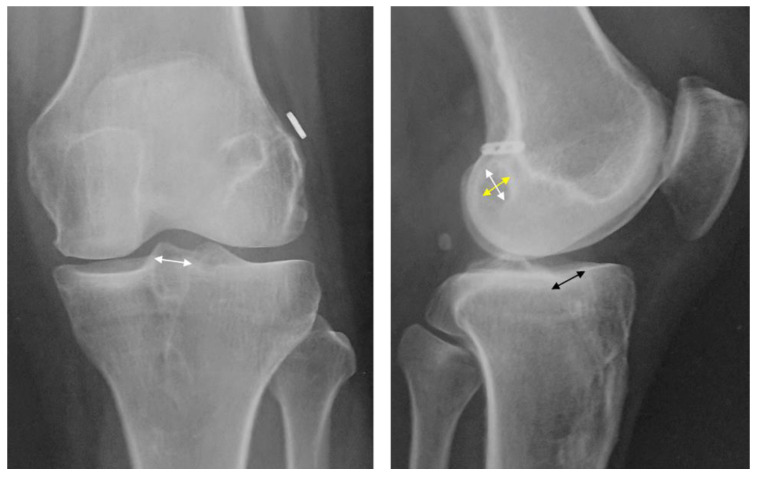
Radiographic findings at the final observation. Bone tunnel enlargement evaluation in the X-ray front view (**left**), the medial-lateral diameter of the tibial bone tunnels at the aperture sites that were between the inner aspect of the sclerotic tunnel wall was measured (white arrow). In the lateral radiograph (**right**), the anterior-posterior diameter (yellow arrow), proximal-distal diameter (white arrow) of the femoral bone tunnel, and anterior-posterior diameter of the tibial bone tunnel (black arrow) at the aperture sites that were between the inner aspect of the sclerotic tunnel wall were measured.

Regarding the bone tunnel enlargement evaluation, the femoral and tibial tunnels at the aperture site were evaluated (Figure 1) [14,15,16]. In the frontal X-ray view, the medial-lateral diameter (MLD) of the tibial bone tunnels at the aperture site, measured between the inner aspect of the sclerotic tunnel wall, was recorded (Figure 1) [19].

The anteroposterior diameter (APD) and proximal-distal diameter (PDD) of the femoral bone tunnel and the APD of the tibial bone tunnels at the aperture sites between the inner aspect of the sclerotic tunnel wall were measured (Figure 1) [19]. If the tunnel walls were not visible, they were excluded from measurements. Moreover, measurements were recorded using the Picture Archiving and Communication System (C@RNACORE, FUJIFILM Medical Co., Tokyo, Japan). The bone tunnel enlargement rates of the APD and PDD of the femoral bone tunnel at the aperture site and the APD and MLD of the tibial bone tunnels at the aperture sites were calculated [14,15,16]. The increase in bone tunnel diameter was calculated as follows: bone tunnel diameter increase rate (%) = ((APD or PDD or MLD at final observation) − (APD or PDD or MLD of the drill diameter at the time of surgery)) × 100/APD or PDD or MLD of the drill diameter at the time of surgery [14,15,16,19]. Bone tunnel enlargement rates were compared between the groups during the final observation. Radiographic evaluations were performed in a blinded manner by a single orthopedic surgeon, with values measured to the first decimal place. The intraclass correlation coefficient of intrarater reliability for the bone tunnel enlargement evaluation was 0.81–0.92, indicating that the measurement accuracy was high.

### 2.3. Statistical Analyses

Participant characteristics between the CaP and control groups were compared using Student’s *t*-test, and Fisher’s exact probability tests were performed between the two groups, the CaP and conventional method groups. Sex, age, and obesity are known risk factors for ACL re-rupture and knee osteoarthritis [20,21,22,23]. The Lysholm scale, Tegner scale, and K–L grade were analyzed using a generalized linear mixed model (GLMM) based on repeated measurement data from preoperative and final observations, with time, group, sex, age, and BMI as fixed effects, and the effect of individual differences as variable effects. In addition, bone tunnel enlargements for the femur and tibia were analyzed using generalized linear models (GLMs) with group, sex, age, and BMI as the main effects. Statistical analysis was performed using IBM SPSS 28.0, with a 5% level of statistical significance. The results are presented as means with 95% confidence intervals (CIs).

A power calculation was performed with a confidence level of 95% (α = 0.05) and a power (1−β) of 80%, resulting in an estimated sample size of 36 patients per group. In a previous report, 73 patients were enrolled, and a statistically significant difference was observed between the groups in terms of bone tunnel enlargement by CT analysis [14]. To account for potential patient dropout, 90 patients were enrolled in the present study [15,16].

## 3. Results

**Figure 2:**
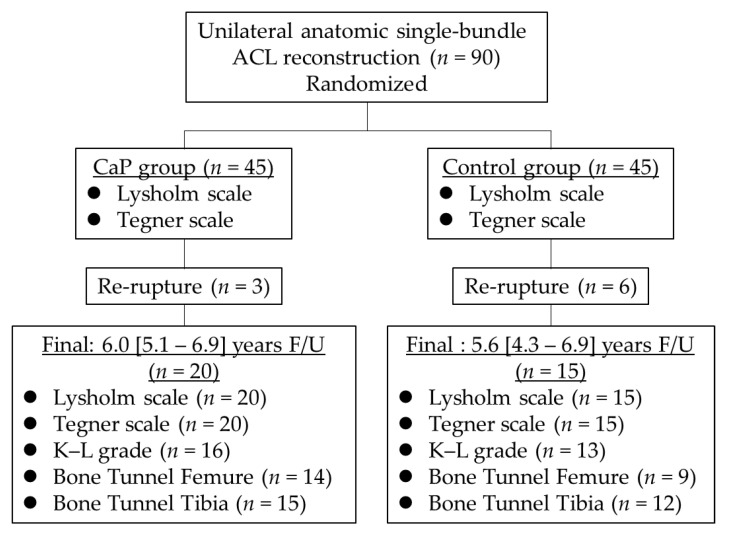
Study flowchart. Patients in the CaP group underwent anatomical single-bundle ACL reconstruction using a calcium phosphate-hybridized graft. Patients in the control group underwent anatomical single-bundle ACL reconstruction with a conventional tendon graft. Patients who could be followed up for >3 years (CaP group, *n* = 20, 6.0 [5.1–6.9] years; control group, *n* = 15, 5.6 [4.3–6.9] years) were retrospectively evaluated in this study. ACL, anterior cruciate ligament; CaP, calcium phosphate; F/U, follow-up; K–L, Kellgren–Lawrence.

A flowchart of the present study is presented in Figure 2. At the time of surgery, the CaP group had 45 patients (age, 27.1 [14–54] years; sex, 21 male and 24 female individuals), and the control group had 45 patients (22.9 [13–58] years; sex, 26 male and 19 female individuals). After identifying patients who were followed up for >3 years, the CaP group included 20 patients (20 knees; average follow-up period, 6.0 [5.1–6.9] years), whereas the control group included 15 patients (15 knees; average follow-up period, 5.6 [4.3–6.9] years) (Table 1).

No significant differences were noted in age, sex, height, BMI, operative findings of meniscal injury, duration from injury to operation, or follow-up period, except for weight, between the groups (Table 1). In the CaP group, 17 patients aged >18 years and three patients aged <18 years, whereas seven and eight patients were aged >18 and <18 years, respectively, in the control group.

The clinical outcomes and K–L grades are summarized in Table 2. A GLMM analysis was performed, considering the two factors of time (i.e., preoperative and final observation) and group (CaP and control groups) as well as confounding factors, such as sex, age, and BMI, are fixed effects, and the individual patient as a random effect.

The Lysholm scale showed a significant fixed effect of time, with higher scores at the final observation than before surgery. However, there were no significant fixed effects between the groups or interactions between the group and time.

Regarding the Tegner scale, there was a significant fixed effect of time, indicating lower scores at the final observation than before surgery. Additionally, there were significant fixed effects between the groups, with the CaP group tending to score lower than the control group.

For the K–L grade, there was a significant fixed effect of time, showing higher scores at the final observation than before surgery. The between-group fixed effect was also significant, with significantly higher scores in the CaP group than in the control group.

Bone tunnel enlargement results are presented in Table 3.

Considering that the weight of the control group was higher than that of the CaP group and the fact that age and sex should have been considered, a GLM analysis was performed. The analysis included the factors of the group (CaP group and control group) and sex, as well as main effects such as age and BMI.

The between-group main effect for an increase in the femoral tunnel APD was significant. Interestingly, the increase was significantly smaller in the CaP group than in the control group. Although the main effect between the groups was not significant for the increase in femoral tunnel PDD, the difference between the two groups was large. The MCID using the receiver operating characteristic curve cut-off value was 15%. However, the value in the CaP group was significantly lower. In contrast, no significant between-group main effect was noted for the APD or MLD of the tibial tunnel. The main effects of sex and the effects of confounding factors, such as age and BMI, were not significant in any of the items.

## 4. Discussion

In this study, the clinical results were similar in both groups. However, the femoral bone tunnel increase rate at more than an average of 5 years of follow-up was lower in the CaP group than in the control group after anatomical single-bundle ACL reconstruction. The osteoarthritis grades progressed in both groups.

In the present study, the CaP-hybridized tendon graft reduced bone tunnel enlargement on the femoral side APD and PDD compared with the corresponding control group at the final observation. After ACL reconstruction with suspensory fixation, the bone tunnels at the joint aperture site are enlarged by bone resorption and are associated with graft tunnel micromotion [24,25], and cytokines in synovial fluid induce delayed tendon-to-bone healing at the joint aperture site of the bone tunnel [26]. In animal studies, the CaP-hybridized tendon graft promoted bone formation on the tendon graft surface in the bone tunnel compared with the untreated tendon graft [11,12]. Similar to the bone, the microstructure of the CaP-hybridized tendon surface is complex and includes type I collagen and calcium phosphate; therefore, osteoblasts and osteoclasts may recognize the CaP-hybridized tendon surface as bone and form a new bone [11,12]. Moreover, the CaP-hybridized tendon graft promoted tendon-to-bone healing. Normal insertion-like structures containing the cartilage layer at the joint aperture site of the femoral and tibial bone tunnels were regenerated [8,13]. It is considered that the normal insertion-like structure containing the cartilage layer may be formed by the influence of tensile and compressive forces applied to the interface after direct bonding between the CaP-hybridized tendon graft and bone tunnel [8,13]. In contrast, it is considered that a conventional tendon graft receives a shearing force (graft tunnel micromotion) between itself and the bone tunnel, and fibrous tissue intervenes at the interface between the bone tunnel and the tendon graft [8,13]. The normal insertion-like structure containing the cartilage layer can play as a shock absorber against graft tunnel micromotion. Furthermore, as the normal insertion-like structure containing the cartilage layer is formed at the joint aperture site, the normal insertion-like structure may prevent an influx of the synovial fluid between the bone tunnel and the tendon graft. The normal insertion-like structure may prevent graft tunnel micromotion. Therefore, the CaP group may have prevented the progression of bone tunnel enlargement at more than an average of 5 years of follow-up. In the present study, the CaP-hybridized tendon graft prevented bone tunnel enlargement, particularly on the femoral side. In a previous study, bone tunnel enlargement was particularly evident on the femoral side in conventional ACL reconstruction [27]. Graft tunnel micromotion can induce greater shear stress at the interface in the femoral bone tunnel than on the tibial side [28].

Excessive tunnel enlargement may necessitate using a two-stage reconstructive approach in revision ACL reconstruction. The initial procedure is necessary to graft bone to the enlargement tunnels, followed by delayed ACL revision surgery [29]; this has a clinically negative impact. The CaP-hybridized tendon graft is also considered advantageous for revision ACL reconstruction as it can prevent bone tunnel enlargement. Revision ACL reconstruction using the CaP-hybridized tendon graft may not require bone grafting and may be performed as a single-stage ACL revision surgery.

Both the CaP group and the control group showed progression in osteoarthritis grade from the preoperative stage to the final observation. However, the CaP group had worse grades compared to the control group. Age had a significant effect on the results, indicating that older patients had worse grades. Although sex and BMI did not show any significant effects, previous studies have noted the occurrence of knee osteoarthritis following ACL reconstruction [2,9]. Microinstability may persist after ACL reconstruction and progress to knee osteoarthritis. In ACL reconstruction, the knee joint is considered to have microinstability because the anchoring point between the tendon graft and bone tunnel is in the bone tunnel, which is far from the joint aperture site [8,13]. The CaP-hybridized tendon graft may prevent graft tunnel micromotion via enhanced tendon-to-bone healing at the joint aperture site. The use of a CaP-hybridized tendon graft is expected to prevent graft tunnel micromotion by promoting better tendon-to-bone healing at the joint aperture site. However, contrary to expectations, the CaP group did not show a reduced progression of osteoarthritis. This could be due to inconsistencies in background factors, such as the difference in osteoarthritis grade between the two groups at the preoperative stage. Thus, future studies should involve longer, background-matched prospective follow-ups with matched backgrounds to investigate the potential of CaP grafts in preventing the progression to knee osteoarthritis.

In biomedical applications, radiation stability against radiation damage of CaP has been reported [30,31,32]. Although radiation stability was not evaluated in this study, the postoperative clinical outcomes in the CaP group were improved compared with those preoperatively and were similar to the results in the control group. Moreover, no adverse events were found during the follow-up period. The Lysholm scale showed no significant effects of sex, age, and BMI. Similarly, the Tegner scale did not show any effects of sex and BMI, although age had a significant effect, with older patients having lower scores. Therefore, the use of CaP-hybridized tendon graft is considered safe for anatomical single-bundle ACL reconstruction. The safety and efficacy of the CaP-hybridized tendon graft have also been described previously [14,15,16]. Although the radiation stability of the CaP-hybridized tendon graft is unknown, its safety and efficacy are considered acceptable. Clinical outcomes, including anterior laxity of the knee after ACL reconstruction, are associated with tunnel enlargement [33]. In contrast, no association was found between clinical outcomes, including anterior knee instability and tunnel enlargement [19]. However, the association between clinical outcomes and tunnel enlargement, particularly anterior knee instability, remains controversial. Therefore, longer follow-up is necessary to investigate the association between bone tunnel enlargement and clinical outcomes using the CaP-hybridized tendon graft in anatomical single-bundle ACL reconstruction.

The intraoperative CaP hybridization method is simple, taking only approximately 10 min, and it can be performed safely. In addition, the CaP-hybridized tendon graft can be used safely without causing postoperative complications. Even after an average of more than 5 years after anatomical single-bundle ACL reconstruction, the CaP-hybridized tendon graft may prevent bone tunnel enlargement more effectively compared to the conventional method. Preventing bone tunnel enlargement may avoid two-stage surgery during revision ACL reconstruction.

However, this study has some limitations. This was a follow-up of a randomized controlled trial with many missing data points and a low follow-up rate. As the postoperative follow-up period for ACL reconstruction is usually 2 years owing to the possibility of re-rupture [14,15,16], the number of patients that continued with the follow-up decreased after that point. In both the groups, no significant effects of sex and BMI were observed in bone tunnel enlargement, K–L grade, Lysholm scale, and Tegner scale. However, there were discrepancies in the level of osteoarthritis, duration from injury to operation, body weight, and age, as they were not well-matched between the groups. Therefore, future studies should focus on conducting prospective studies with better background matching. Another limitation is that bone tunnel evaluation in this study was performed using plain radiography instead of CT, although it did not contradict previous reports on bone tunnel enlargement using CT [14,15,16]. There were only a few evaluation items for the clinical outcomes. Finally, considering the short follow-up period of this study, longer follow-ups are required for more comprehensive insights.

## 5. Conclusions

The findings of this study indicate that anatomical single-bundle ACL reconstruction using the CaP-hybridized tendon graft is safe for clinical practice. Moreover, the use of CaP-hybridized tendon grafts can effectively reduce femoral bone tunnel enlargement over an average follow-up period of more than 5 years. Prospective longer follow-up studies are necessary to reveal the clinical effects of the CaP-hybridized tendon grafts in anatomical single-bundle ACL reconstruction.

## Figures and Tables

**Table 1:** Patient profiles at the final observation.

	CaP Group (*n* = 20)	Control Group (*n* = 15)	*p* Value
Age (years)	32.0 (24.4–37.5)	25.9 (19.0–32.9)	0.165
Sex (male/female)	8/12	10/5	0.189
Height (cm)	165.1 (161.8–168.4)	169.5 (165.4–173.6)	0.104
Weight (kg)	62.1 (57.0–67.2)	70.4 (64.8–76.0)	0.041 *
BMI (kg/m^2^)	22.7 (21.2–24.2)	24.5 (22.6–26.5)	0.142
Operative findings of meniscal injury (MM/LM)	7/7	4/5	0.269
Duration from injury to operation (months)	15.1 (2.6–27.6)	2.6 (1.5–3.8)	0.082
Follow-up period (years)	6.0 (5.1–6.9)	5.6 (4.3–6.9)	0.764

The analysis was performed using Student’s *t*-test and Fisher’s exact probability test. The results are presented as means and 95% confidence intervals. * *p* < 0.05: significant difference between both groups. BMI, body mass index; CaP, calcium phosphate; LM, lateral meniscus; MM, medial meniscus.

**Table 2:** Clinical outcomes at the final observation.

	Preoperative (Preinjury)		Final		Fixed Effect						Model Fit Criteria		
	CaP Group	Conventional Method	CaP Group	Conventional Method	Time	Group	Time × Group	Sex	Age	BMI	AICc	BIC	MCID
Lysholm scale	54.5 (50.5–58.5)	49.2 (44.5–53.9)	95.5 (91.5–99.5)	97.0 (92.3–101.7)	<0.001	0.095	0.111	0.873	0.548	0.206	487.287	491.374	9.23
Tegner scale	5.9 (5.4–6.5)	6.9 (6.2–7.6)	5.5 (5.0–6.1)	5.9 (5.2–6.6)	0.012	0.049	0.239	0.459	<0.001	0.811	239.875	243.961	0.70
K–L grade	1.1 (0.8–1.41)	0.4 (0.1–0.8)	1.8 (1.5–2.1)	1.2 (0.8–1.5)	<0.001	0.015	0.686	0.571	0.002	0.150	112.622	116.236	0.33

The analysis was performed using a generalized linear mixed model. Results are presented as means and 95% confidence intervals. For the fixed effects, *p*-values were shown. MCID was calculated using the standard error of change for the CaP group. AIC, Akaike’s information criterion; BIC, Bayesian information criterion; BMI, body mass index; CaP, calcium phosphate; K–L, Kellgren–Lawrence; MCID, minimal clinically important difference.

**Table 3:** Results of bone tunnel enlargement.

	Group		Main Effect				Model Fit Criteria		
	CaP Group	Control Method	Group	Sex	Age	BMI	AIC	BIC	MCID
Femur									
APD (%)	7.9 (−1.1–16.8)	29.2 (17.9–40.5)	0.004	0.708	0.927	0.284	207.398	214.211	16.05
PDD (%)	7.9 (−1.9–17.8)	22.8 (10.9–34.7)	0.062	0.936	0.668	0.819	201.346	207.892	15.00
Tibia									
APD (%)	8.9 (−3.0–20.8)	22.9 (10.0–35.8)	0.128	0.219	0.764	0.781	246.473	254.022	18.88
MLD (%)	21.0 (13.5–28.6)	13.6 (5.1–22.2)	0.217	0.142	0.455	0.253	233.175	240.950	22.25

The analysis was performed using generalized linear models. Results are presented as the means and 95% confidence intervals. The main effects showed *p*-values. MCID was calculated using ROC curves. AIC, Akaike’s information criterion; APD, anteroposterior diameter; BIC, Bayesian information criterion; BMI, body mass index; CaP, calcium phosphate; MCID, minimal clinically important difference; MLD, medial-lateral diameter; PDD, proximal-distal diameter.

## Data Availability

The datasets generated and/or analyzed during the current study are available from the corresponding author upon reasonable request.
